# Management of Chronic Seromas and the Role of Bleomycin Sclerotherapy: A Two-Case Report

**DOI:** 10.7759/cureus.100746

**Published:** 2026-01-04

**Authors:** Volkan Kiper, Berat Acu, Fatih Aksüt, Sipan Zir Yıldırım, Aydan A Köse

**Affiliations:** 1 Department of Plastic and Reconstructive Surgery, Eskişehir Osmangazi University Hospital, Eskişehir, TUR; 2 Department of Interventional Radiology, Eskişehir Osmangazi University Hospital, Eskişehir, TUR

**Keywords:** bleomycin, chronic seroma, morel lavallee syndrome, persistent seroma, sclerosant, sclerotherapy

## Abstract

Seroma is a common postoperative complication after several plastic surgery operations. They mostly resolve on their own, but from time to time, they can persist and become chronic. This article reports two cases of persistent seromas, a post-abdominoplasty seroma and a Morel-Lavallée lesion, successfully treated with intralesional bleomycin and reviews their etiology, prevention, and treatment with a focus on sclerotherapy. The different choices of sclerosants are discussed with their mechanism of action, focusing on bleomycin, which was used in two different cases of persistent seromas successfully without complication. To our knowledge, this article is the first to report the clinical use of bleomycin as a sclerosant for the treatment of seroma.

## Introduction

Seroma is a collection of serous fluid in a dead space, containing blood plasma and lymph fluid [1]. It forms due to damage to vascular and lymphatic structures in the subcutaneous tissue during surgery [2]. Clinically significant symptomatic seromas occur mostly after breast cancer surgeries, abdominal wall surgeries, especially abdominoplasty, post-bariatric body contouring, latissimus dorsi muscle harvest, and axillary and inguinal lymph node dissections [1,3,4]. Seromas may develop even after a local excision of a lipoma because the main reason for the serous fluid accumulation is pouch formation [4]. Therefore, seroma mainly occurs as a complication of an operation, but it may form as a result of high-energy trauma as well, which is known as a Morel-Lavallée lesion. Morel-Lavallée lesions are closed injuries characterized by degloving of the superficial soft tissues from the underlying fascial layers [5]. These lesions are mostly found over the greater trochanter of the femur and are typically unilateral. They are also reported in the lumbar, prepatellar, scapular, buttock, and trunk regions [5].

Whether traumatic or postoperative, transected blood vessels and lymphatic channels lead to the accumulation of serous fluid in a surgically or traumatically created anatomical dead space [6]. Seromas usually resolve on their own, but in some cases, they can be resistant and may persist despite multiple aspirations. In these cases, sclerotherapy may be an appropriate option for the treatment. This article presents two patients treated with intralesional bleomycin sclerotherapy after several failed aspiration attempts.

## Case presentation

Presentation of the first case

**Figure 1:**
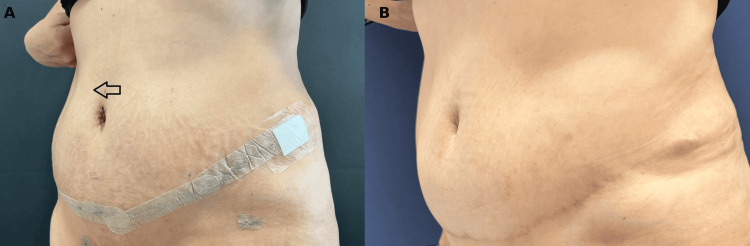
Resolution of postoperative seroma following intralesional bleomycin sclerotherapy (A) On postoperative day 12, the patient presented with a subcutaneous mass beneath the abdominoplasty flap following physical activity beyond postoperative recommendations. (B) One-year postoperative follow-up demonstrated complete resolution of the seroma following the treatment, which included intralesional bleomycin sclerotherapy.

A 48-year-old, healthy female patient underwent combined abdominoplasty and liposuction. Quilting sutures were applied, and drains were inserted under the abdominoplasty flap during the operation. External support was applied using an abdominal binder and corset. The postoperative period was uneventful. The drain was removed on the postoperative fifth day, and the patient was discharged. On the postoperative twelfth day, after hard physical activity beyond recommendations, she felt an enlarging palpable subcutaneous mass (Figure 1A). Immediate ultrasonography showed a hematoma beneath the abdominoplasty flap. Under sterile conditions, 440 cc of serohemorrhagic fluid was aspirated, and a closed suction drain was inserted. When the daily output ceased at under 30 cc after two weeks, the drain was removed. However, the seroma recurred. Several drainage attempts didn’t help eliminate the seroma cavity. Therefore, intralesional sclerotherapy with bleomycin was decided upon. Fifty cc of hemorrhagic fluid was aspirated, 15 mg bleomycin mixed with 15 ml of prilocaine was injected inside the cavity, and cold compression was applied after the procedure. The follow-up ultrasonography revealed the complete resolution of the seroma (Figure 1B). No complications of bleomycin were seen in the patient.

Presentation of the second case

**Figure 2:**
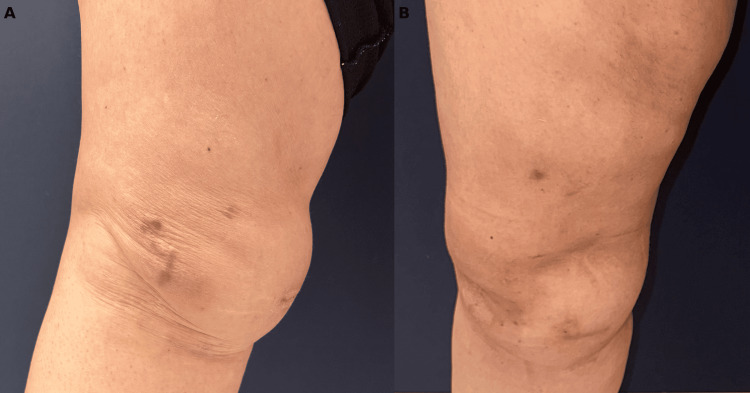
Resolution of a chronic Morel-Lavallée lesion following intralesional bleomycin sclerotherapy (A) At the first presentation, a large, long-standing, fluctuating subcutaneous mass was found spreading over her right inner and anterior thigh. (B) Complete resolution of the chronic Morel-Lavallée lesion was observed after the intralesional bleomycin injections.

A 36-year-old female patient presented with a large seroma pouch on her right leg (Figure 2A). She had a history of a vehicle accident four months ago. She was followed with closed suction drainage and referred to our facility due to the persistence of fluid accumulation. Physical examination showed a fluctuating mass spreading over her right inner and anterior thigh. The patient was diagnosed with a Morel-Lavallée lesion. Ultrasonographic evaluation revealed a widely distributed anechoic collection, extending from the proximal region of the right thigh along the anteromedial section of the thigh and reaching the knee joint. It was compressed between the subcutaneous tissue and the superficial fascia of the rectus femoris, vastus medialis, and gracilis muscles. The patient was referred to the interventional radiology department, where an ultrasonography-guided intralesional bleomycin injection procedure was performed after 190 cc of serous fluid removal. One month later, the seroma cavity was reduced in size but not completely resolved. Fifteen mg of bleomycin mixed with 15 ml of prilocaine was injected into the cavity after 100 cc of serous fluid aspiration. Following this injection, the seroma fully regressed (Figure 2B). No complications related to bleomycin were observed.

## Discussion

Seromas usually resolve spontaneously, but in large seromas, spontaneous resolution may take time, cause patient discomfort, and lead to the prolongation of the hospital stay. Also, they are associated with delayed wound healing, an increased risk of flap necrosis, and carry the risk of becoming infected and present as an abscess [2,4,7].

In order to prevent seroma formation, closed-suction drains should be inserted after the surgical procedures in which a dead space is created and should not be removed until the daily output falls under 20-30 cc. It is proven that volume-controlled removal is much more effective than time-controlled removal. Instead of using electrocautery, sharp dissection, with which a surgeon can manage to preserve the vascular and lymphatic integrity better, provides lower seroma rates [4]. In addition, there are other risk factors associated with patients, such as hypertension and obesity [8,9].

Mobile surgical sites slow down tissue adhesion and the obliteration of the dead spaces. For instance, constant chest movements during respiration for the breast region and shoulder movements for the axillary region create shear forces that prevent flap adhesion [7]. As a result, during the postoperative period, it is important to instruct the patient to keep the surgical area as immobile as possible. Also, it is helpful to use quilting or progressive tension sutures during the operation, which immobilize the surgical site without preventing the postoperative ambulation of the patient [4].

Compression bandages, such as abdominal binders, are traditionally used in the postoperative period to prevent seroma formation. Nevertheless, studies showed that the use of compression dressing is not effective for the prevention of postoperative seroma formation [10-12]. O’Hea et al. observed that compression dressing didn’t reduce the seroma formation [12]; on the contrary, the incidence of seroma formation increased.

Sclerosants are being used for the treatment of seromas, but the prophylactic use of sclerosants during the operation resulted in a significant increase in seroma formation. Despite their effectiveness in treating seromas, they have no role in preventing them [4].

The management of recalcitrant seromas starts with screening by magnetic resonance imaging (MRI) or ultrasonography (US). The initial approach is usually repeated aspiration every two to three days with the use of a compression bandage. Large and chronic seromas, which do not respond to multiple aspirations, require advanced methods ranging from excision of the cavity surgically, capsulectomy, to the injection of a sclerosant agent [3,6,13].

Sclerosant injection induces fibrosis for the purpose of obliterating the dead space [6]. The use of sclerosant agents, especially doxycycline and talc, is common in cases of pericardial effusion and malignant pleural effusion [14]. However, it has recently entered into the treatment algorithms of the recalcitrant seroma in the last two decades.

Several published reports document the successful use of various types of sclerotherapy for the treatment of recalcitrant seromas. A systematic review reports a success rate of 95.7% for the sclerotherapy on chronic seromas [15]. Suggested sclerosant agents are doxycycline, erythromycin, bleomycin, vancomycin, ethanol, povidone iodine, tetracycline, and talc [2,5,6,16,17]. Even though it is shown that all of these agents are effective with low complication rates, there is still a lack of comparative randomized controlled studies for the comparison of the efficacy and safety of different options of sclerosant agents. Thus, there is no consensus on the use of the optimal sclerosant agent or the treatment algorithm in our daily practice yet.

Most sclerosant agents, such as erythromycin and tetracycline, stimulate the formation of fibrous tissue by initiating an inflammatory process. Doxycycline destroys the mesothelial cells that surround the pseudocyst. Ethanol causes protein coagulation and hyperosmolar cell destruction, which leads to tissue necrosis and fibrosis [6].

There are a few sclerosants, which stimulate tissue adhesion without an inflammatory response. Fibrin glue, which contains fibrinogen, factor XIII, thrombin, and calcium, stimulates the clotting cascade. Produced fibrin sticks tissue surfaces together. Talc (hydrated magnesium silicate) is frequently used in the treatment of seromas, but the mechanism of its effect is still not well-understood. Talc stimulates a fibrotic reaction, which involves polymorphonucleocytes, cytokines, interleukin 8 (IL-8), and fibroblast growth factors (FGF). However, animal experiments showed that the talc injection doesn’t produce any active inflammatory process. OK-432 is a low virulence strain of *Streptococcus pyogenes,* which also treats seromas without producing an inflammatory response [2,6,18].

Bleomycin was initially used as an anti-tumor agent by inhibiting DNA synthesis. Its sclerosing effect on endothelial cells through an inflammatory reaction was demonstrated later. Its first use as a sclerosant agent was for the treatment of lymphangioma. Observed adverse effects of bleomycin sclerotherapy are cutaneous and include local swelling and hyperpigmentation [19,20]. Bleomycin as a sclerosant agent has fewer side effects than the other agents, but it should be kept in mind that bleomycin can cause pulmonary fibrosis, which was reported when it is administered intravenously as a chemotherapy agent with much higher doses [16]. Despite its high efficacy with low complication rates as a sclerosant agent, the main disadvantage of bleomycin is that it has a slightly higher price compared to other sclerosants [14,18].

Bleomycin is frequently used in malignant pleural and pericardial effusions, but it’s not commonly used for the treatment of seroma. An animal study showed that bleomycin has a significant effect on reducing seroma formation after mastectomy and axillary lymph node dissection with a very low complication rate, and it is recommended for clinical use [9].

## Conclusions

In our study, we report the successful use of bleomycin as a sclerosing agent for the management of intractable seroma, with no complications observed during or after treatment. However, the limited patient size and retrospective nature of the study are the primary limitations of this study. Prospective studies with larger cohorts and comparative analyses including other sclerosant agents are needed to validate the efficacy and safety of bleomycin.
